# Differential responses of the soil microbial community in two pitaya orchards with different mulch types

**DOI:** 10.1038/s41598-019-46920-3

**Published:** 2019-07-18

**Authors:** Juan Luo, Min Xu, Zhao Qi, Rui Xiong, Yu Cheng, Chengli Liu, Shuangshuang Wei, Hua Tang

**Affiliations:** 1 Hainan Key Laboratory for Sustainable Utilization of Tropical Bioresources, College of Tropical Crops, Hainan University, No. 58 Renmin Avenue, Haikou, 570228 Hainan P.R. China; 2 University of Sanya, No. 191 Yingbin Avenue Xueyuan Road, Sanya, 572000 Hainan P.R. China

**Keywords:** Microbial ecology, Bacterial genomics, Fungal genomics

## Abstract

To prevent plants from being damaged due to extreme temperature and sunlight, the pitaya orchards in Hainan Province, China, are increasingly adopting living and black fabric cloth mulching. In this study, an Illumina Hiseq sequencer was employed to compare the soil microbial communities of two pitaya orchards, one covered by living mulching (LM) and the other covered by black fabric cloth (FC). Bacterial abundance was higher in the LM orchard than in the FC orchard (1.19 × 10^4^ *versus* 4.49 × 10^4^ g^−1^ soil). In contrast, fungal abundance was higher in the FC orchard than in the LM orchard (2.71 × 10^6^ *versus* 2.97 × 10^5^ g^−1^ soil). We also found that the most dominant species in the FC orchard were from the genus *Neoscytalidium*, which included species that could cause infection in a large variety of plant hosts. However, the LM orchard mainly harbored useful fungal species, such as *Trichoderma* and *Chaetomium*. Soil nutrients were positively correlated in the FC orchard, which potentially indicated that the FC orchard could demonstrate better fertilizer utilization efficiency. However, the LM and FC strategies have both advantages and disadvantages with regards to the cultivation management of pitaya orchards.

## Introduction

Pitaya cultivation plays an essential role in the economic and social importance of Hainan Province, China. In these areas, agricultural soils are exposed to sun and heat for prolonged periods of time; this can result in damage to the shallow roots of pitaya plants. Mulching, one of the most widely used field management techniques in local orchards, is an effective method with which to control soil temperature, reduce soil evaporation and increase root growth^1–4^.

In recent years, living mulching (LM) and black fabric cloth mulching (FC) have been increasingly adopted by orchards in South China to protect the roots of fruit trees or to prevent soil degradation. These methods represent the main management strategies in pitaya orchards as they encourage the growth of fruit trees. LM is a promising farming system which is being used more widely in South China orchards^5^. This is an effective method with which to increase or maintain crop yield by improving the soil environment^6–8^. LM prevents soil evaporation, increases soil humidity and temperature, and improves the growth of flowers and fruits^7,9^.

FC can increase soil respiration to a greater extent than non-mulching treatment^10–13^ and increases CO_2_ emission by affecting the soil gas concentration gradient in the soil profile. FC also reduces the use of herbicides and fertilizers by reducing weed and pest pressure^14,15^. Other studies have suggested that continuous black fabric cloth mulching can increase soil bulk density and increase soil pH, which has a negative effect on the growth of plants^16^.

In South China, the roots of pitaya trees are easily affected by extremes of temperature and damaging sunlight. One possible way to resolve this issue is to cover the ground surface with different types of mulch. However, there is no agreement on which mulching patterns are better for the health of soil in orchards.

Over recent years, LM and black FC have become the most commonly used management strategy in the pitaya orchards of South China. We therefore chose two nearby pitaya orchards, one covered by LM and the other covered by FC. The diversity and relative abundance of microbial communities were then determined by analyzing the 16S rRNA gene of bacteria, and the 18S rRNA gene of fungi, using Illumina HiSeq technology.

## Results

### Physicochemical analysis

The soil fertility of FC in Liguo orchard was consistently better than LM in Fulin orchard. The content of available phosphorus (AP) ranged from 179.75 mg/kg to 419.20 mg/kg and decreased in the following order: FC1 > FC2 > FC4 > FC5 > FC3 > LM1 > LM5 > LM3 > LM2 > LM4; There was a significant difference (*p* < 0.05) between the two orchards in this respect. The mean available potassium (AK) content of the FC soil (280.47 mg/kg) was significantly higher (*p* < 0.05) than that of the LM soil (94.33 mg/kg) (Table 1). The AP and AK content of the FC soil were approximately 88.36% and 192.01% higher than that of the LM soil, respectively. The content of alkali-hydrolyzable nitrogen (AN) (68.99 mg/kg in FC and 78.54 mg/kg in LM, respectively) showed no significant difference (*p* > 0.05) when compared between the two orchards. Soil organic carbon (OC) ranged from 5.20 g/kg to 8.48 mg/kg; there was no significant difference (*p* > 0.05) between the two orchards in this respect. Soil pH ranged from 4.34 to 6.66, and was significantly lower (*p* < 0.05) in LM soil than in FC soil (Table 1).

**Table 1:** The concentrations of available nitrogen (AN), available phosphate (AP), available potassium (AK), soil organic carbon (OC) and pH in two pitaya orchards (Liguo and Fulin).

	AN mg/kg	AP mg/kg	AK mg/kg	OC g/kg	PH
	Mean	Mean	Mean	Mean	Mean
FC1	69.3 ± 1.74	419.2 ± 14.17	370.50 ± 6.52	8.40 ± 0.5	5.39 ± 0.42
FC2	60.06 ± 2.61	359.5 ± 22.79	230.35 ± 19.63	6.78 ± 0.58	5.61 ± 0.3
FC3	84.7 ± 1.47	307.75 ± 7.47	250.62 ± 5.92	7.53 ± 0.55	6.23 ± 0.16
FC4	47.74 ± 1.07	332.5 ± 9.34	250.87 ± 17.27	6.97 ± 0.13	6.66 ± 0.33
FC5	83.16 ± 1.52	327.00 ± 9.57	300.02 ± 9.58	7.75 ± 0.29	5.83 ± 0.14
**Mean value of FC group**	**68.99a**	**349.19a**	**280.47a**	**7.49a**	**5.94a**
LM1	70.84 ± 0.46	242.50 ± 0.64	76.32 ± 1.26	8.48 ± 0.08	4.43 ± 0.23
LM2	56.98 ± 1.83	179.75 ± 0.9	88.33 ± 1.16	5.20 ± 0.18	4.34 ± 0.41
LM3	138.60 ± 0.40	188.00 ± 1.13	86.37 ± 1.13	6.22 ± 0.17	4.21 ± 0.25
LM4	77.00 ± 0.60	173.25 ± 1.1	104.49 ± 1.38	5.98 ± 0.32	4.71 ± 0.12
LM5	49.28 ± 0.08	211.75 ± 1.08	116.12 ± 1.05	8.48 ± 0.28	4.36 ± 0.27
**Mean value of LM group**	**78.54a**	**199.05b**	**94.33b**	**6.87a**	**4.41b**

Within the same column, significant differences are indicated by different letters (*P* < 0.05). FC1, FC2, FC3, FC4 and FC5 represent the five replicates from the Liguo orchard (FC group: Liguo orchard with black fabric cloth mulching); LM1, LM2, LM3, LM4 and LM5 represent the five replicates from the Fulin orchards (LM group: Fulin orchard with living mulching).

### The richness and diversity of bacterial and fungal communities

On average, sequencing yielded 78564 ± 4695 sequences per sample for bacteria, and 85689 ± 4572 sequences per sample for fungi. Low quality reads were then filtered out. The screened DNA sequences were then clustered into 111810 and 7304 identity operational taxonomic units (OTUs) at a 95% sequence similarity threshold for bacteria and fungi, respectively. Next, we compared OTU numbers between LM and CM; only bacteria showed a statistically significant difference (*P* < 0.05) (Table 2). The coverage of the samples ranged from 83.94 to 99.86%. The Chao1 species richness index was significantly higher in LM than that in FC (*P* < 0.05); while the Shannon index was significantly higher in FC than LM (*P* < 0.05), but there were individual exceptions for bacteria (for example, the value of FC4 was lower than most of the stations) (Table 2).

**Table 2:** Estimates of bacterial and fungal α-diversity in two pitaya orchards (Liguo and Fulin).

sites	Bacteria					Fungi				
	Total reads	OTUs (97%)	Chao 1 (97%)	Shannon (97%)	Coverage (97%)	Total reads	OTUs (97%)	Chao 1 (97%)	Shannon (97%)	Coverage (97%)
LM1	59,908	15,433	53,291	7.30	85.83	80,733	655	1,188	2.24	99.61
LM2	48,784	6,169	19,460	6.36	94.54	80,328	228	493	0.91	99.86
LM3	61,036	13,764	45,156	7.06	88.01	91,717	699	1,442	2.28	99.57
LM4	65,443	18,499	64,426	7.65	83.94	89,487	831	1,508	2.40	99.50
LM5	61,848	15,112	48,841	7.08	87.01	90,864	848	1,613	2.51	99.52
**LM group**	**59,404a**	**13,795a**	**46,234a**	**7.09a**	**87.87b**	**86,226a**	**652a**	**1,249a**	**2.07b**	**99.61a**
FC1	5,4764	7155	21,927	6.91	94.21	82,467	1,558	3,120	4.95	97.79
FC2	6,391	8,841	19,960	7.50	94.61	85,286	874	1,899	3.99	99.27
FC3	63,540	11,207	29,586	7.75	91.79	83,765	551	1,137	3.22	99.57
FC4	57,469	7,746	16,662	7.59	94.80	90,745	239	445	2.20	99.79
FC5	61,598	7,884	19,011	7.29	94.56	81,507	821	1,613	3.59	99.23
**FC group**	**60,352a**	**8,566b**	**21,429b**	**7.41a**	**93.99a**	**84,754a**	**808a**	**1,643a**	**3.59a**	**99.13a**

Within the same column, significant differences are indicated by different letters (*P* < 0.05). FC1, FC2, FC3, FC4 and FC5 represent five replicates from the Liguo orchard (FC group: Liguo orchard with black fabric cloth mulching); LM1, LM2, LM3, LM4 and LM5 represent five replicates from the Fulin orchard (LM group: Fulin orchards with living mulching); OTUs: operational taxonomic units.

### The abundance of bacterial and fungal communities

**Figure 1:**
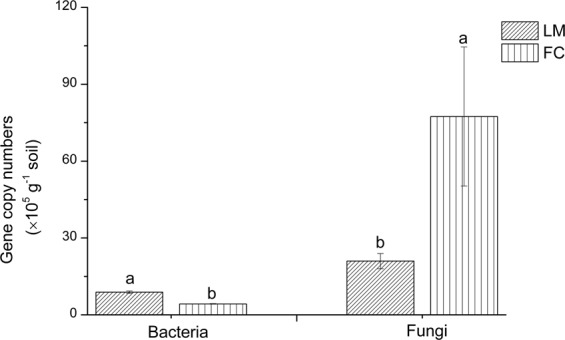
Abundance of bacteria and fungi per microgram of fresh soil in FC (Fulin orchard with black fabric cloth mulching) and LM (Liguo orchard with living mulching). Error bars indicate two standard errors.

The copy numbers of bacteria and fungi in the two orchards were significantly different. The results of real-time polymerase chain reaction (PCR) showed that bacterial abundance in FC soil was significantly lower (*P* < 0.05) than that in LM soil and averaged 4.26 × 10^5^ and 8.86 × 10^5^ 16S rRNA gene copies/g fresh soil, respectively. ITS genes were approximately 1–2 orders of magnitude more abundant than the 16S rRNA gene. Unlike bacteria, the gene copy numbers of ITS in FC were 7.74 × 10^6^ gene copies/g fresh soil, which was significantly greater (*P* < 0.05) than that in LM soil (2.09 × 10^6^ gene copies/g fresh soil) (Fig. 1).

**Figure 2:**
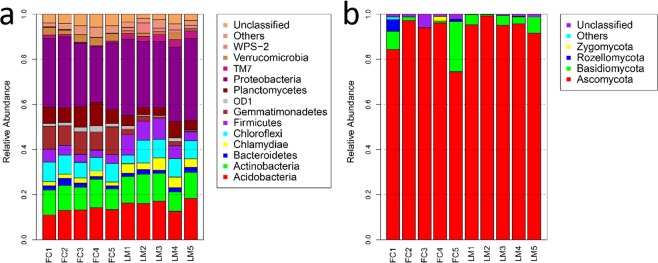
Taxonomic distributions of bacterial phyla (**a**) and fungal phyla (**b**) at 10 sites (FC1, FC2, FC3, FC4 and FC5 represent five replicates from Liguo orchards with black fabric cloth mulching; LM1, LM2, LM3, LM4 and LM5 represent five replicates from Fulin orchards with living mulching).

At the phylum level of bacteria, the dominant taxa among the 10 samples were *Proteobacteria* (30.80%), *Acidobacteria* (14.54%), *Actinobacteria* (11.86%), *Chloroflexi* (7.69%), *Planctomycetes* (6.10%), *Firmicutes* (5.67%), *Gemmatimonadetes* (5.64%), *Chlamydiae* (3.16%), *Verrucomicrobia* (2.32%), TM7 (1.89%), *Bacteroidetes* (1.85%), OD1 (1.37%) and WPS-2 (1.16%). Furthermore, *proteobacteria* was the predominant bacterial phylum, representing 28.98% of the bacterial population of FC soil and 32.57% of LM soil (Fig. 2a). *Gemmatimonadetes* was significantly more abundant in FC soil compared with LM soil, however TM7 and WPS-2 were more abundant in LM soil than FC soil. At the order level, *Rhodospirillales*, *Acidobacteriales*, *Bacillales* and *Xanthomonadales* were highly abundant in both FC and LM soils. *Rhodospirillales-*affiliated sequences were more abundant in LM than in FC soil, and *Gaiellales* and *Bacillales* were more abundant in FC soil compared to LM soil (Fig. S1).

The dominant fungal phyla, *Ascomycota*, was present in 94.50% of the 10 samples (Fig. 2b). At the order level, *Hypocreales*, *Sordariales*, *Botryosphaeriales*, *Eurotiales*, *Incertae_sedis, Pezizales* and *Tremellales* were present in 49.82%, 14.73%, 9.93%, 7.94%, 2.71%, 2.62% and 2.57% of samples, respectively (Fig. S1). Among the samples, *Hypocreales* and *Sordariales* were significantly more abundant in LM soils compared to FC soils. In contrast, *Botryosphaeriales* was significantly more abundant in FC soil than in LM soil.

### Hierarchical cluster analyses

**Figure 3:**
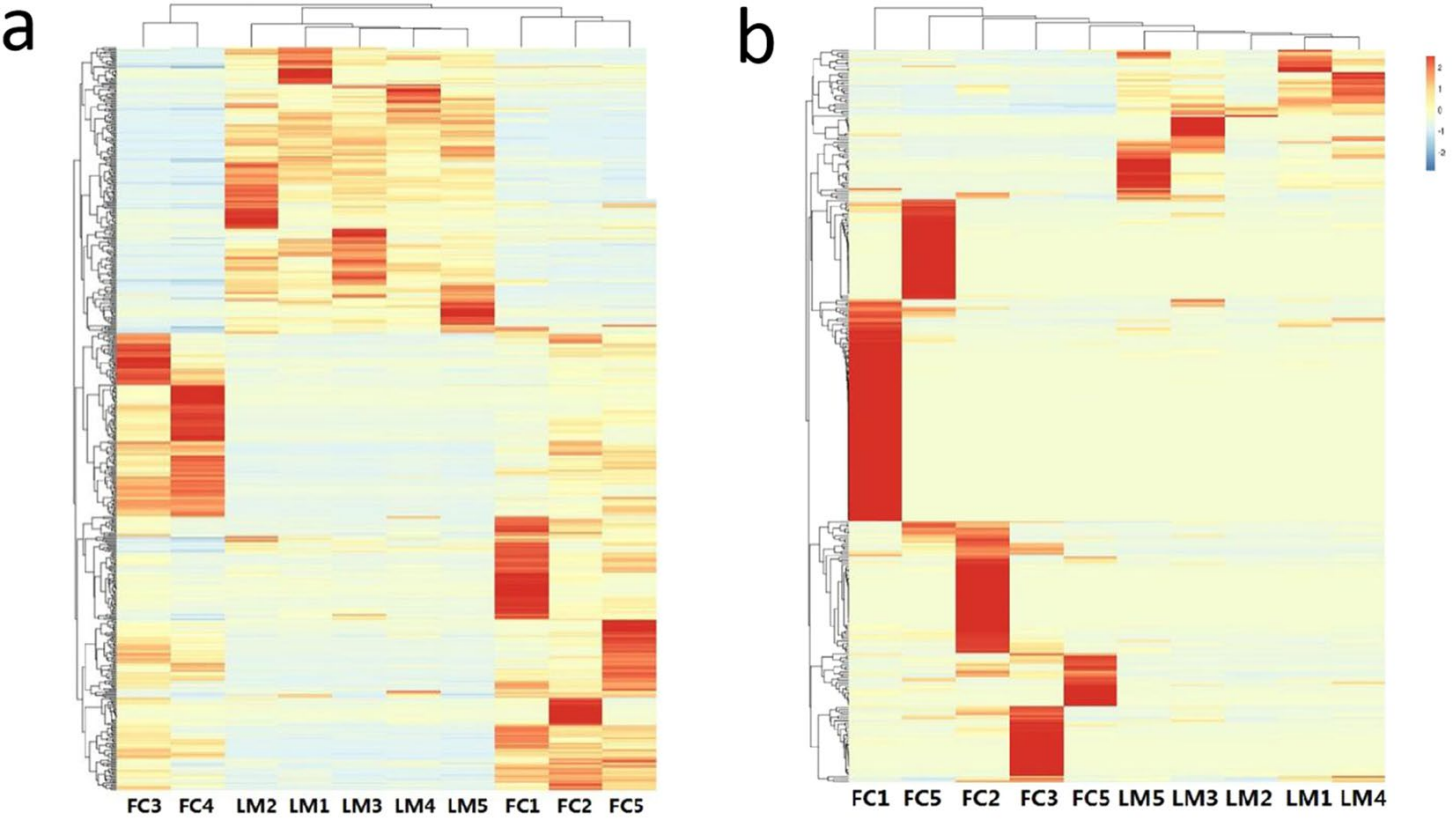
Hierarchical cluster analysis based on abundance of the OTUs identified in each sample in bacterial (**a**) and fungal (**b**) data-sets. The color intensity in each panel shows the level of enrichment in the samples, blue to red representing lower to higher levels of enrichment.

Hierarchically-clustered heatmap analysis was used to analyze the relative abundance of bacterial and fungal OTUs. We used OTUs which included tag numbers representing more than 1% of the total numbers. Results showed that for bacteria, the five samples of LM were grouped together; however, the FC samples were divided into two groups: FC3 and FC4 were grouped together and were distinct from FC1, FC2 and FC5. For fungi, samples from LM and FC were grouped together. These results showed that the diversity of OTUs in FC was higher than in LM, and that the LM soil had a certain effect on the soil bacterial and fungi community structure (Fig. 3).

### Links between selected soil fertility properties and bacterial and fungal taxa

**Figure 4:**
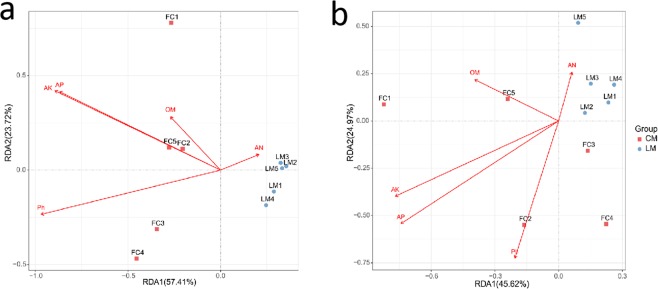
Redundancy analysis (RDA) based on the bacterial (**a**) and fungal (**b**) community composition and soil characteristics. Abbreviations: soil organic carbon (OC), available phosphate (AP), available potassium (AK) and alkali-hydrolyzable nitrogen (AN).

The effect of soil fertility on microbial community composition was performed by redundancy analysis (RDA). Analysis of the bacterial community showed that the first two axes of RDA explained 53.41% and 23.72% of the total variation, respectively. The first component (RDA1) explained 53.41% of the total variation. The first two axes of RDA explained 45.62% and 24.97% of the total variation in the fungal data. Results showed that pH, AK, AP and OC were positively correlated with FC, and negatively correlated with LM. In contrast, AN was positively correlated with LM (Fig. 4).

## Discussion

Living and cloth mulching are widely used in orchards to protect the roots of fruit trees, or to suppress weeds. In this study, we measured the nutrient levels in two orchards, one covered by weeds and the other covered by black fabric cloth. The results showed that soil AP and AK were significantly higher in FC than in LM. Previous studies in apple orchards showed that the soil in film mulch contained a higher content of AK than in grass mulch, which is in accord with the present results^17^. Mulching with plastic film was previously shown to significantly increase AP content^18^, while soil exposed to living mulching had a lower AP content than soil without mulching^19^. Another study showed that the AP content was higher in living mulch than in film mulch, and therefore differed from our present results^17^. Our current results may show that cloth film, rather than living mulch, has a more significant impact on strengthening ground surface stability, which helps to stabilize soluble minerals.

The V3/V4 region which was the most accurate and widely used in the field of deep sequencing^20^ and the ITS2 region which has lower GC variation, shorter length and greater taxonomic information content^21^ were used for Illumina sequencing studies on bacterial and fungal communities separately. In the present research, bacterial alpha-diversity and richness were higher in the LM orchard than in the FC orchard. Previous research has clearly indicated that living grass mulching or film mulching exerts an obvious influence upon the diversity and community composition of soil bacteria^17,20,22–25^; these earlier results were consistent with our present research. For example, Jiao *et al*.^26^ and Qian *et al*.^27^ both found that living mulch management could significantly improve bacterial diversity in apple orchards. Living mulching treatment, which showed higher bacterial community diversity, may also contribute to the carbon-cycling process, along with N concentration and availability^26,28^.

Some studies have found that plant type can influence bacterial community structure and diversity via root zone effects and root exudates^29–31^. Compared to non-mulched treatment, black fabric cloth mulching has been reported to increase the population of soil bacteria and fungi remarkably^32–35^. Previous research has shown that fabric cloth mulch could help enhance the preservation of soil warming, moisture and fertility^35–37^, which helps to promote the decomposition of organic matter and soil microbial activity^3^. Bacterial and fungal biomass was also found to significantly increase organic cover to a greater extent than either black polyethylene mulch or non-mulch^37,38^.

For bacteria, *Phylogenetic* analysis showed that in FC soil, the five major *phylogenetic* groups were *Proteobacteria (*29.00%), *Acidobacteria* (12.93%), *Actinobacteria* (10.75%), *Gemmatimonadetes* (9.86%) and *Planctomycetes* (7.78%). In LM soil, the five major *phylogenetic* groups were *Proteobacteria* (32.57%), *Acidobacteria* (16.09%), *Actinobacteria* (11.41%), *Chloroflexi* (7.67%) and *Firmicutes* (7.18%) (Fig. S2). *Proteobacteria*, *Acidobacteria* and *Actinobacteria* which are the most common phyla in both LM and FC, and are widely distributed in most soil types^39^.

*Gemmatimonadetes* and *Planctomycetes* were more abundant in FC soil than in LM soil. In contrast, *Chloroflexi* were more abundant in LM than FC soil, as also reported in previous research^17^. Dong *et al*.^40^ observed a higher abundance of *Actinobacteria* in autumn mulching (AM) treatment than in non-mulching (NM) samples. Among the fungi, the most dominant species in FC soil were unclassified (23.35%), *Neoscytalidium* (20.97%), *Penicillium* (10.47%) and *Chaetomium* (10.29%); *Trichoderma* and *Chaetomium* were most abundant in LM soil, accounting for 67.18% and 13.32%, respectively (Fig. S2).

For FC, the most dominant species were the genus *Neoscytalidium*, which includes species that could cause infection in a large variety of plant hosts including saprophytes, parasites and endophytes^41^. *Penicillium* species, which are ubiquitous soil fungi that are especially found in moderate climates^42^, were more abundant in FC than LM soil. In a previous study, Wenyi *et al*. also found that *Penicillium* increased significantly in plastic film mulching compared to non-mulching, which is in accord with the present research^40^. Some species of *Penicillium* play an important role in the promotion of plant growth by enhancing stress tolerance or by improving the utilization of phosphorus in plants^43,44^. *Chaetomium* species, one of the largest genera of *saprobic ascomycetes*, were abundant in both orchards^45^. The most common species of fungi in LM soil were *Trichoderma*, with an average contribution of 67.18%. *Trichoderma* species, which are usually found in soil and root ecosystems, could be used for root colonization to promote root growth, control disease and absorb nutrients^46^.

The influence of five major soil characteristics on the distribution and diversity of bacteria and fungi were analyzed by RDA. These results indicated a positive correlation between AP, AK, pH and FC; AP, AK and pH, which were significantly higher in FC than in LM (Table 1), have been shown to have a significant effect on soil bacterial and fungal composition under different mulching applications. These results showed that mulching might cause different chemical structure in decomposing plant residues, or cause a shift in microbial history strategies^40^.

In the two orchards tested here, the cultivation management strategies were very similar, except for the different mulching protocols. Therefore, we speculate that the primary factor affecting the species, numbers and diversity of microorganisms were the mulching patterns. LM and FC, could affect the moisture, temperature, and physicochemical properties of the soil^47^, which would then affect the diversity and numbers of bacteria and fungi. For example, the abundance of AP in FC soil allows *Penicillium* species to thrive and expand, because *Penicillium* can improve the utilization of phosphorus. In addition, microorganisms influence soil nutrients by decomposing organic material or by participating in carbon and nitrogen cycling^48,49^.

## Conclusion

In general, the abundance and diversity of microorganisms in the two types of pitaya orchard soil were distinct from each other; the cause of these differences was the differing mulching strategy. Gene copy numbers of the 18S rRNA were greater than that of 16S rRNA in the two orchards, and the abundance and diversity of fungi was higher in FC than in LM. However, *Neoscytalidium* was the dominant species in FC, which may imply that the plants with FC were more vulnerable to soil root disease. On the other hand, the positive correlation between AP, AK, pH and FC is perhaps a sign that FC can lead to better fertilizer utilization efficiency. Although fungi was less abundant in LM than those in FC, LM soil mainly harbors useful fungal species. However, bacteria species were more abundant in LM than in FC. To summarize, both LM and FC have their advantages and disadvantages; the two different mulching patterns can be mixed to some extent in order to improve the integrated quality of soil.

## Materials and Methods

### Experimental site and experimental design

The experimental field is located in Ledong city (latitude 18°31′ to 18°32′ N and longitude 108°54′ to 108°55′ E), Hainan Province, China. This region has a tropical oceanic monsoon climate, with an average yearly sunshine duration of 2090.8 hours, an average annual temperature of 25 °C and a mean annual precipitation of more than 1500 mm; these conditions are highly favorable for the growth of pitaya trees.

In this region, we chose two adjacent pitaya orchards (Fulin orchard and Liguo orchard) which are nearly 2.5 km apart and located to the side of a highway. The soils of the two orchards are coastal sand soil and pitaya trees (the Dahong variety – ‘red heart’) have grown in these orchards for more than 3.5 years. The mulching treatments used in the two orchards are different. The Fulin orchard was covered by LM while the Liguo orchard was covered by FC. In terms of LM treatment, self-seeding weeds (mainly graminaceous) were grown freely in-rows and inter-rows, and were mowed at least twice a year. The FC treatment involved a black fabric cloth placed in the rows. The other farming methods used in the two orchards are fairly consistent: the row-column distance is 1.8–2.2 × 1.4–1.6 m; every 4–5 pitaya trees are supported by concrete square columns measuring 10 cm × 10 cm × 150 cm and there is 0.8 m between columns and 1.5 m between rows. Furthermore, 2.25 × 105 kg/ha^−1^ of organic fertilizer is provided once a year, and 300 kg/ha^−1^ of compound fertilizer is used once a month (N:15%, P_2_O5:15%, K_2_O:15%). Chemical fertilizer (N:10%, P_2_O5:40%, K_2_O:10%) is routinely applied as a top dressing 2–3 times a month.

### Soil sampling and DNA extraction

We collected five parallel samples which were selected in a “W” pattern (about 0.5 m away from surrounding streams); each sample was homogeneously mixed by three soil cores at a depth of 0–15 cm in the root environments within a 1 m × 1 m quadrat. Approximately 600 g of each soil sample was homogenized and subdivided, and then placed into sterile labeled plastic bags. A sub-sample was stored at −80 °C for DNA extraction, and the other sub-sample was dried and sieved for chemical analysis. All of the soil samples were collected in one day.

Total genomic DNA was extracted from 0.5 g of material per soil sample using the PowerSoil DNA extraction kit (Mo Bio Laboratories, Carlsbad, CA, USA) in accordance with the manufacturer’s instructions. The concentration of the extracted DNA was then quantified using a NanoDrop 2000 UV-Vis Spectrophotometer (NanoDrop Products, Wilmington, DE, USA).

### Soil chemical properties and nutrient content

The soil samples used for the analysis of chemical properties were dried in a drying oven at 105 °C for 24 h in order to determine soil water content. The dried soil was first sieved by a 2 mm mesh, and the other soil characteristics were then measured as follows: Soil pH was measured using a glass electrode in distilled water-to-sieved soil ratio of 2.5:1. Alkali-hydrolyzable nitrogen (AN) was measured by alkaline hydrolysis diffusion. Available phosphorus (AP) was determined using the Bray II method. Available potassium (AK) was determined with a flow auto-analyzer. Soil organic carbon (OC) was measured using the K_2_CrO_7_-H_2_SO_4_ oxidation method^50^.

### High-throughput sequencing

The V3 + V4 region of 16S rRNA, and the ITS2 region of the 18S rRNA, were then amplified in order to construct bacterial and fungal community libraries, respectively, using tag high-throughput sequencing. The primers 341 F (5′-CCTACGGGNGGCWGCAG-3′) and 805 R (5′-GACTACHVGGGT ATCTAATCC-3′) were used to target the V3 + V4 hypervariable region of the 16S rRNA gene. The forward primer BITS (5′-ACCTGCGGARGGATCA-3′) and reverse primer B58S3 (5′-GAGATCCRTTGYTRAAAGTT-3′) were used to target the ITS2 region of the 18S rRNA gene^51,52^.

The PCR reaction was performed in a 25 μL reaction mixture which contained 1 μL of template DNA, 15 μL of 2 × Taq PCR MasterMix (Aidlab), 0.5 μL of forward and reverse primers (5 μM of each primer) and 8 μL of double distilled water. Thermal cycling conditions included an initial denaturation at 94 °C for 45 s, 30 cycles of 95 °C for 30 s, 54 °C for 30 s, and 72 °C for 30 s; this was followed by an indefinite period at 14 °C. Negative and positive control samples were used in each reaction to monitor for potential contamination. PCR products were extracted and purified, then pooled at equal concentrations and diluted to create one sample. The sequencing libraries were prepared using the TruSeqDNA PCR-Free Sample Preparation Kit (Illumina, USA), and index barcodes were added. The libraries were then used for sequencing on the Illumina Hiseq PE250 platform at Personal Biotechnology Co., Ltd. Shanghai, China.

### Data analysis

The raw sequencing reads generated from the Hiseq PE250 system were assembled and quality-filtered using the “Quantitative Insights into Microbial Ecology”(QIIME) software package (Version 1.7.0)^52,53^. After low quality reads had been filtered out, sequences with 97% similarity were assigned using Uparse (Version 7.0.1001) to the same operational taxonomic units (OTUs). Then, the Greengenes Datebase^54^ was used to analyze the phylogenetic affiliation for each representative sequence with RDP Classifier (http://rdp.cme.msu.edu). The taxonomic alpha diversity, species coverage (Coverage), species richness (Chao) and species diversity (Shannon Index) were calculated using Mothur software (v. 1. 30. 1)^55^.

Correlation between soil characteristics and soil samples were determined by Redundancy analysis (RDA) using CANOCO software^54^. Heatmaps were generated for the relative abundance of OTUs using R package (version 2.15; The R Project for Statistical Computing, http://www.R-project.prg).

One-way analysis of variance (ANOVA), followed by the least significant difference (LSD) test (with 95% confidence levels), was used to examine differences between the two orchards. Statistical analyses were performed with SPSS software version 19.0 for windows (SPSS Inc., Chicago, IL, USA).

Finally, the sequences in this study were submitted to the Sequence Read Archive (SRA) database (NCBI) under accession number SRP160993 for bacteria and SRP 162183 for fungi.

### Quantitative PCR

The copy numbers of the 16S and the 18S rRNA gene in the soil samples was determined using the quantitative PCR (qPCR) system. The primers 341 F (5′-CCTACGGGAGGCAGCAG-3′) and 534 R (5′-ATTACCGCGGCTGCTTGG-3′) were used to target 16s rRNA genes^51^, and the primers ITS1-f (5′-TCCGTAGGTGAACCTGCGC-3′) and 5.8 S (5′-TCCTCCGCTTATTGATATGC-3′) were used to target ITS genes^56^.

The qPCR reactions involved 0.3 μL of each primer (0.4 mM), 12.5 μL of SYBR Premix Ex TaqTM (Takara, Dalian, China), 0.3 μL of ROX dye, 1.0 μL of template DNA; double-distilled water was added to create a final volume of 20 μL. Each reaction was duplicated and included negative samples (free of DNA) to monitor for potential contamination. The assays were carried out on an ABI 7000 (PE Applied Biosystems) real-time PCR machine and involved a thermal profile of 95 °C for 5 min followed by 35 cycles of 95 °C for 15 s, 55 °C for 30 s, 72 °C for 1.5 min, and a plate reading step at 80.5 °C for 30 s. For evaluating amplification specificity, the PCR products were subjected to melt curve analysis. The standard curves for qPCR was determined by cloned genes which were serially diluted over five orders of magnitude.

## Supplementary information

Supplementary information
