# Supplementary material for: Differential responses of the soil microbial community in two pitaya orchards with different mulch types
Source: Sci Rep. 2019 Jul 18;9:10413. doi: 10.1038/s41598-019-46920-3 (PMC6639319; doi:10.1038/s41598-019-46920-3)
Supplement: Supplementary file 1 — Supplementary information [file 41598_2019_46920_MOESM1_ESM.pdf]

Differential responses of the soil microbial community in two pitaya orchards with different mulch types

Juan Luo[1,2], Min Xu[1], Zhao Qi[1], Rui Xiong[1], Yu Cheng[1], Chengli Liu[1], Shuangshuang Wei[1] and Hua Tang[1*]

[1]Hainan Key Laboratory for Sustainable Utilization of Tropical Bioresources, Institute of Tropical Agriculture and Forestry, Hainan University, No.58 Renmin Avenue, Haikou 570228, Hainan, P. R. China.
[2]University of Sanya, No.191 Yingbin Avenue Xueyuan Road, Sanya 572000, Hainan, P. R. China.

*Corresponding author:
Hua Tang
Hainan Key Laboratory for Sustainable Utilization of Tropical Bioresources, Institute of Tropical Agriculture and Forestry, Hainan University, No.58 Renmin Avenue, Haikou 570228, Hainan, P. R. China
E-mail address: thtiger@163.com.
Telephone: 13138915025

Supplementary Figure

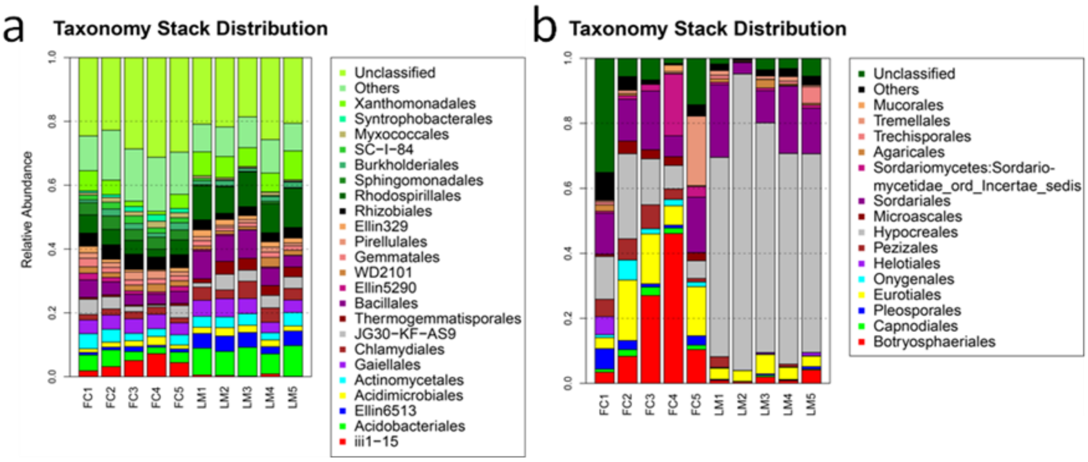

Fig. S1 Taxonomic distributions of bacterial order (a) and fungal order (b) in 10 sites (FC1, FC2, FC3, FC4 and FC5 represent the five replicates of Liguo orchards with plastic cloth mulching; LM1, LM2, LM3, LM4 and LM5 represent the five replicates of Fulin orchards with living mulching)

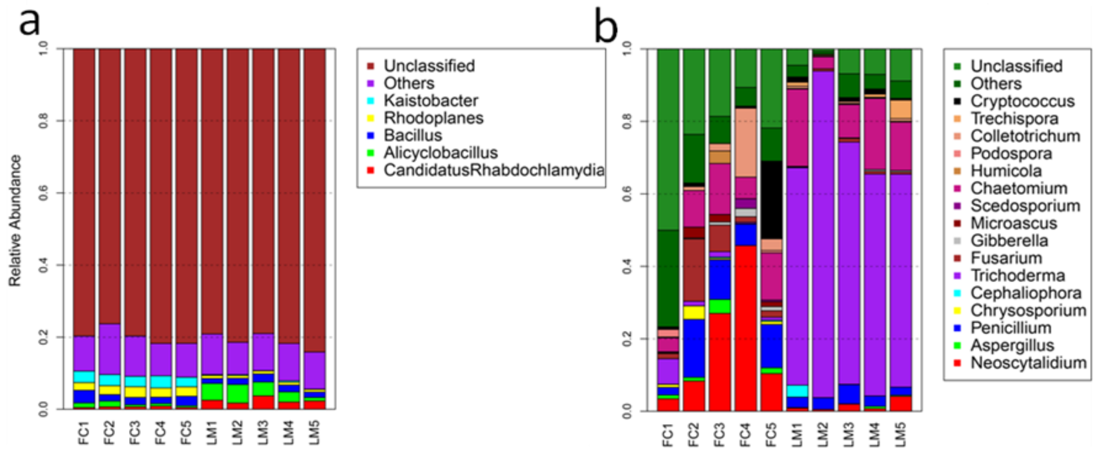

Fig. S2 Taxonomic distributions of bacterial genus (a) and fungal genus (b) in 10 sites (FC1, FC2, FC3, FC4 and FC5 represent the five replicates of Liguo orchards with plastic cloth mulching; LM1, LM2, LM3, LM4 and LM5 represent the five replicates of Fulin orchards with living mulching)
